# Periodontitis relates to benign prostatic hyperplasia via the gut microbiota and fecal metabolome

**DOI:** 10.3389/fmicb.2023.1280628

**Published:** 2023-12-13

**Authors:** Xing-Pei Guo, Jun Yang, Lan Wu, Cheng Fang, Jia-Min Gu, Fei Li, Han-Song Liu, Lu-Yao Li, Shuang-Ying Wang

**Affiliations:** ^1^Department of General Surgery, Zhengzhou Central Hospital Affiliated to Zhengzhou University, Zhengzhou, Henan, China; ^2^Center for Evidence-Based and Translational Medicine, Zhongnan Hospital of Wuhan University, Wuhan, China; ^3^Department of Urology, The First People's Hospital of Tianmen in Hubei Province, The Affiliated Hospital of Hubei University of Science and Technology, Tianmen, China; ^4^Department of Stomatology, Zhongnan Hospital of Wuhan University, Wuhan, China; ^5^Department of Urology, Zhongnan Hospital of Wuhan University, Wuhan, China

**Keywords:** periodontitis, benign prostatic hyperplasia, gut microbiota, fecal metabolites, multi-omics analysis

## Abstract

**Objectives:**

Periodontitis is associated with benign prostatic hyperplasia (BPH), whether it related to gut floramicrobiota and metabonomics is unclear.

**Methods:**

We established ligature-induced periodontitis (EP), testosterone-induced BPH, and composite rat models. Fecal samples were collected to detect gut microbiota by 16S rDNA sequencing and metabonomics were detected by liquid chromatography tandem mass spectrometry (LC-MS/MS).

**Results:**

Sequencing results revealed differential gut floramicrobiota composition between EP+BPH group and other three groups. The abundances of *Ruminococcus flavefaciens* were significantly increased in EP+BPH group compared with other groups. *Tenericutes*, *Mollicutes, RF39* and *Ruminococcus gnavus* were significantly decreased in EP+BPH group compared with BPH group, while *Ruminococcus callidus* and Escherichia were significantly decreased compared with EP group. For gut metabonomics, LC-MS/MS showed that fecal metabolites and seven metabolic pathways were changed in EP+BPH group, such as biosynthesis of unsaturated fatty acids, steroid hormone biosynthesis. Correlation analysis showed that the alterations of gut metabolism were significantly correlated with differential gut floramicrobiota, such as *Ruminococcus callidus* and *Ruminococcus flavefaciens*.

**Conclusion:**

Our study highlights the relationship of periodontitis and BPH, the alterations of gut floramicrobiota and metabolites may be involved in two diseases, which provides new idea for prevention and treatment of patients with periodontitis concurrent BPH.

## 1 Introduction

Periodontitis is a chronic inflammation of periodontal tissue caused by local factors, with oral microbiota being a common cause of inflammation (Hajishengallis and Chavakis, 2021). Bones and teeth can be damaged as periodontitis progresses, thus becoming a major cause of dentition defects in older adults (Tonetti et al., 2018). In addition, periodontal disease has been identified as a risk factor for a variety of systemic diseases and has an important impact on health (Wu et al., 2019; Zhao et al., 2019; Kapila, 2021). A total of 1.1 billion people worldwide suffered from severe periodontitis, according to the study of the Global Burden of Disease in 2019 (Chen et al., 2021; Luo et al., 2021). Benign prostatic hyperplasia (BPH) is one of the most common urinary system diseases in middle-aged and elderly men, mainly manifested as progressive lower urinary tract symptoms (Bauer et al., 2021). It also has an impact on patients’ quality of life, sexual function, and genitourinary health (Zeng et al., 2022). It is increasing with the aging of the global population and seriously affects men’s health and quality of life (Zhu et al., 2021).

In a previous study involving 2,171 participants, we found that periodontitis significantly increases the risk of BPH, and periodontal disease is positively associated with an increased risk of BPH (Wu et al., 2019). Researchers have detected the same bacteria in subgingival plaque and prostatic fluid in patients with periodontitis and BPH (Estemalik et al., 2017). The two diseases have several common risk factors, such as age, metabolic disorders, and psychological factors (Zhao et al., 2020; Fang et al., 2021). Our studies have found that both periodontitis and BPH were linked with gut microbiota and fecal metabolites (Li et al., 2022; Wu et al., 2022). Oxidative stress and inflammation may play a role in the association between two diseases, and we have come up with an oral–prostate axis hypothesis in which periodontitis may interact with prostatic disease in direct or indirect ways (Fang et al., 2021). Gut microbiota, as a general term for a microbial community residing in the human intestinal tract, has been found to be associated with a variety of diseases with the deepening of research in recent years (Clemente et al., 2012; Lynch and Pedersen, 2016; Hou et al., 2022). We proposed an interaction between periodontitis and BPH, and the interaction may be related to gut microbiota.

In this study, we constructed a complex animal model of periodontitis and BPH through rat experiments. The multiomics method was used by 16S sequencing combined with LC–MS/MS. We have explored the alteration of gut microbiota and fecal metabolites under the interaction of periodontitis and BPH. It was found that the interaction between the two diseases might be related to the diversity and abundance of gut microbiota, fecal metabolites, and metabolic pathways. In addition, alteration of the gut microbiota was associated with fecal metabolites. Our study proposed a potential mechanism of the interaction between periodontitis and BPH, provided ideas for research on the interaction between different diseases, and new strategy for the diagnosis and treatment of periodontitis concurrent with BPH.

## 2 Materials and methods

### 2.1 Animals and experimental design

The male SPF-grade Sprague–Dawley (SD) rats (7 weeks old, n = 20) weighting 300–350 g were provided by the Beijing Vital River Laboratory Animal Technology Co. Ltd. Rats were housed for 1 week under conditions of constant humidity (55 ± 10)%, temperature (22 ± 2)°C and a 12-h light/dark cycle with unrestricted access to water and food. All animal protocols were approved by the Institutional Animal Care and Use Committee (IACUC) of Wuhan University (IACUC animal approval protocol #2018119).

Rats were randomized and divided into four groups (n = 5/group): healthy control group (control), ligature-induced periodontitis (EP), testosterone-induced BPH (BPH), and composite rat models (EP + BPH). The rats in the control group received no intervention. The rats in the EP group underwent ligation of sterile nylon thread around the cervical of bilateral maxillary first and second molars (Hajishengallis and Chavakis, 2021). The rats in the BPH group were anesthetized and castrated. A week later, testosterone (5 mg/kg) was administered to the rats in the BPH group once a day for 4 weeks (Sudeep et al., 2019). The rats in the EP + BPH group were constructed by the above two modeling methods. Rats were weighed and placed on a sterile workbench. The feces were collected in sterile cryopreservation tubes from each rat after the intervention, then labeled and stored at −80°C.

The experimental procedures were operated according to our previous study (Fang et al., 2021).

### 2.2 DNA extraction and 16S rDNA sequencing

Microbiota DNA was extracted using MagPure Stool DNA KF kit B (Magen, China). DNA was quantified with a Qubit fluorometer by using the Qubit dsDNA BR Assay Kit (Invitrogen, United States). 16S rDNA amplicon (V3–V4 region) sequencing was performed on the Illumina HiSeq 2,500 platform (BGI, Shenzhen, China). The primers were all processed with the Illumina standard method, and the sequence used was 341F (50-ACTCCTACGGGAGGCAGCAG-30) and 806R (50-GGACTACHVGGGTWTCTAAT-30). A measure of 30 ng of qualified genomic DNA samples and corresponding fusion primers were taken to configure the PCR reaction system. The PCR products were purified and eluted with AMPure XP beads to construct a library.

### 2.3 LC–MS/MS analysis

Waters 2D UPLC (Waters, Milford, CT, United States) tandem Q Exactive HF high-resolution mass spectrometer (Thermo Fisher Scientific, Waltham, MA, United States) was used to separate and detect metabolites. Waters ACQUITY UPLC BEH C18 column (1.7 μm, 2.1 mm × 100 mm, Waters, USA) was used for chromatographic separation. Q Exactive HF mass spectrometer (Thermo Fisher Scientific, United States) was used for primary and secondary mass spectrometry data acquisition. The samples were analyzed in positive ion (spray voltage was 3.8 kV) and negative ion (spray voltage was 3.2 kV) modes. The mass scanning range was 70–1,050 m/z. The flow rates of sheath gas and auxiliary gas were set to 40 L/min and 10 L/min, respectively.

### 2.4 Bioinformatic analysis and visualization

Compound Discoverer 3.1 (Thermo Fisher Scientific, United States) was used to process raw data and identify metabolites. The R software package metaX (Wen et al., 2017) was used for data processing and analysis. We used principal component analysis (PCA) and partial least-squares method-discriminant analysis (PLS-DA) to analyze the overall distribution and stability among groups. PICRUST2 was used to predict the function of gut microbe bacterial communities (Douglas et al., 2020). The Kyoto Encyclopedia of Genes and Genomes (KEGG) was used for pathway analysis. KEGG pathway enrichment analysis was performed using MBROLE 2.0 (López-Ibáñez et al., 2016). Spearman’s rank correlation coefficient was used to analyze the correlation between microbiota and metabolites and for visualization through heat maps.

### 2.5 Statistical analysis

The data were expressed as means ± standard error of mean (SEM). Statistical analysis was performed using SPSS 17.0 software (SPSS, Inc., Chicago, IL, United States). Analysis of Variance (ANOVA) was used to analyze differences among four groups, and least-significant difference (LSD) was further used for multiple testing. Correlations were identified by Spearman’s rank correlation coefficient. GraphPad Prism v8.0 (GraphPad Software Inc., San Diego, CA, United States) and R are used for various analyses and mapping. A *p-value* of <0.05 was considered significant.

## 3 Results

### 3.1 Overview of 16S rDNA microbiome sequencing

**Figure 1:**
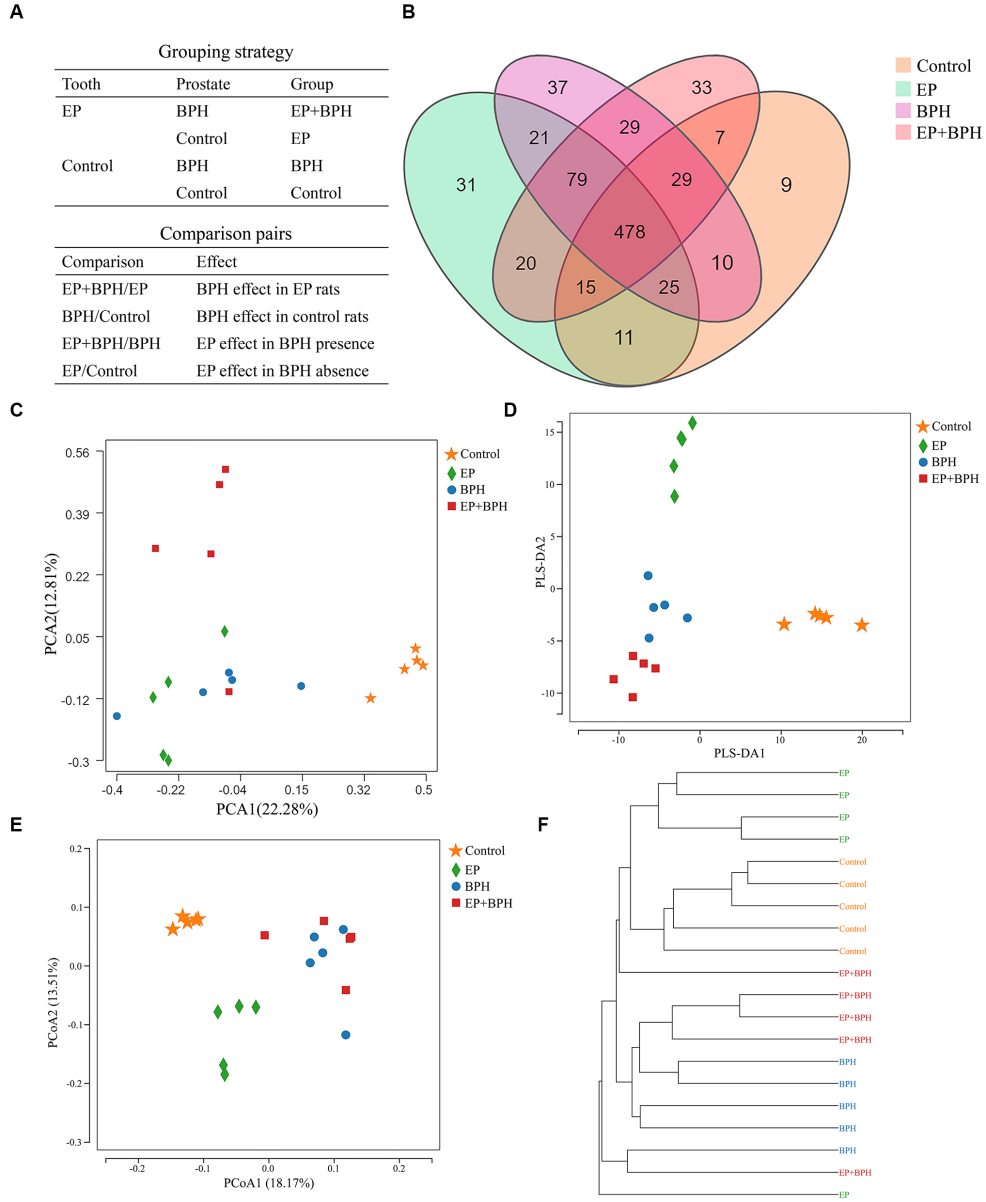
Effect of EP + BPH on the gut microbiota was different from that of EP or BPH **(A)** Grouping and comparison strategies: we divided all the rats into four groups (control group, EP group, BPH group, and EP + BPH group) and compared between groups. BPH/Control represents the effect of BPH on healthy control rats, EP/Control represents the effect of EP on healthy control rats, EP + BPH/EP represents the effect of BPH on EP + BPH rats, and EP + BPH/BPH represents the effect of EP on EP + BPH rats. **(B)** Overview of OTUs in different groups: different colors represent different groups, and the overlapping parts of the numbers are common. Principal component analysis (PCA) **(C)**, partial least squares discrimination analysis (PLS-DA) **(D),** and principal co-ordinates analysis (PCoA) **(E)** for different groups: different colors represent different groups. **(F)** The clustering tree of the unweighted pair-group method with arithmetic means (UPGMA): for samples under the same branch, the shorter the branch length between samples, the more similar the two samples are, and the higher the similarity of species composition is.

Figure 1A shows the grouping and comparison strategies. The OTU rank abundance curve was wide and falling gently, displaying excellent abundance and evenness (Supplementary Figure S1A). Furthermore, the rarefaction curves for all samples tended to be stable, and the number of qualified sequences reached 50,000, indicating that the depth and quantity of sequences met the demands for sequencing and analysis and covered most of the diversity (Supplementary Figure S1B).

The OTU data for four groups suggest differences in gut microbiota between groups. A total of 834 OTUs have been detected by clustering the clean reads with a threshold of 97%, as shown in the Venn diagram (Figure 1B). EP, BPH, EP + BPH, and control groups had 478 common OTUs; 31 OTUs were unique in the EP group; 37 OTUs were unique in the BPH group, 33 OTUs were unique in the EP + BPH group; and 9 OTUs were unique in the control group. PCA of the OTU analysis scatter plot showed that gut microbiota in the EP + BPH group was significantly different from that in the other three groups (Figure 1C), suggesting that EP + BPH alters the structure and diversity of gut microbiota compared to EP, BPH, and control.

### 3.2 Ep + BPH affects the diversity of gut microbiota

Alpha-diversity reflects the species richness and microbiota diversity of individual samples. We estimated gut microbial richness and diversity using the Chao index and the Shannon index, respectively. The analysis showed that compared with the EP group, the EP + BPH group had slightly lower abundance and higher diversity, and compared with the BPH group, the EP + BPH group had slightly higher abundance and lower diversity (*p* > 0.05, Supplementary Figures S1C,D).

PLS-DA analysis scatter plot showed that the EP + BPH group was well separated from the other three groups, suggesting that the gut microbiota of the EP + BPH group was significantly different from other groups (Figure 1D). Principal coordinate analysis (PCoA) and the UniFrac cluster tree represented structural differences in gut microbiota between groups. We found that the BPH group and EP + BPH group were mostly clustered together, while the EP group and control group were almost self-contained (Figures 1E,F), which further indicated that EP and BPH may have an effect on gut microbiota through interaction.

### 3.3 EP + BPH influences composition and functional pathways of gut microbiota

**Figure 2:**
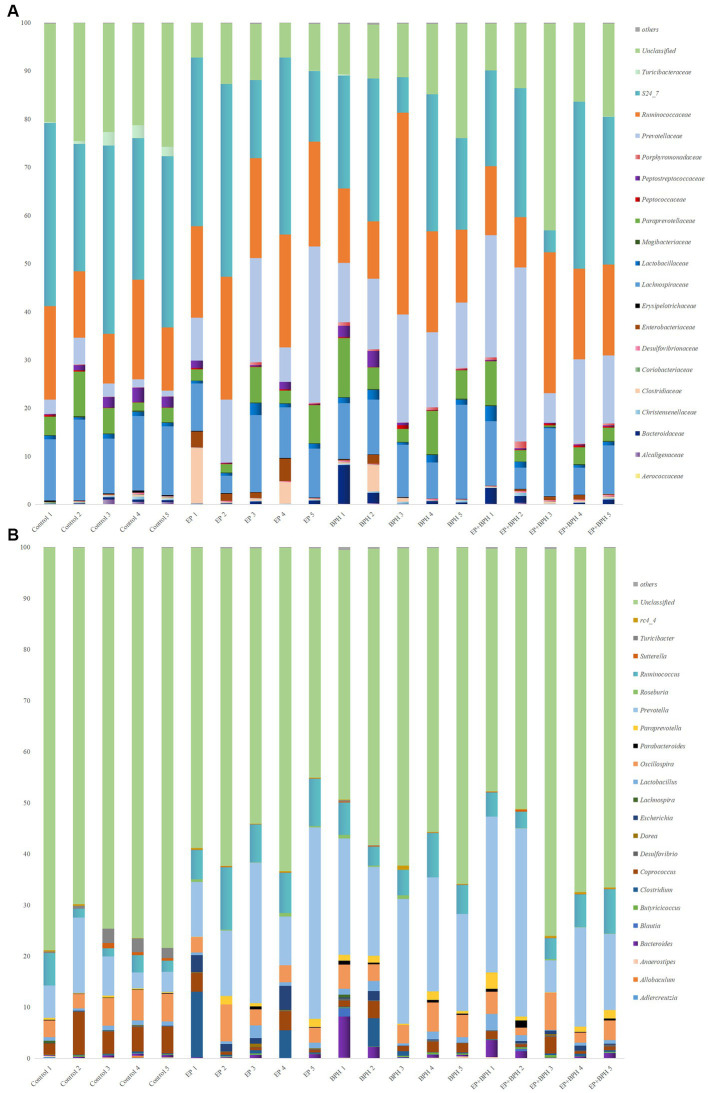
EP + BPH affects the structure of gut microbiota. The species bar chart shows the species composition and proportion of each sample at family level **(A)** and genus level **(B)**: The X axis is the sample name, and the Y axis is the relative abundance of annotated microbiota species.

**Figure 3:**
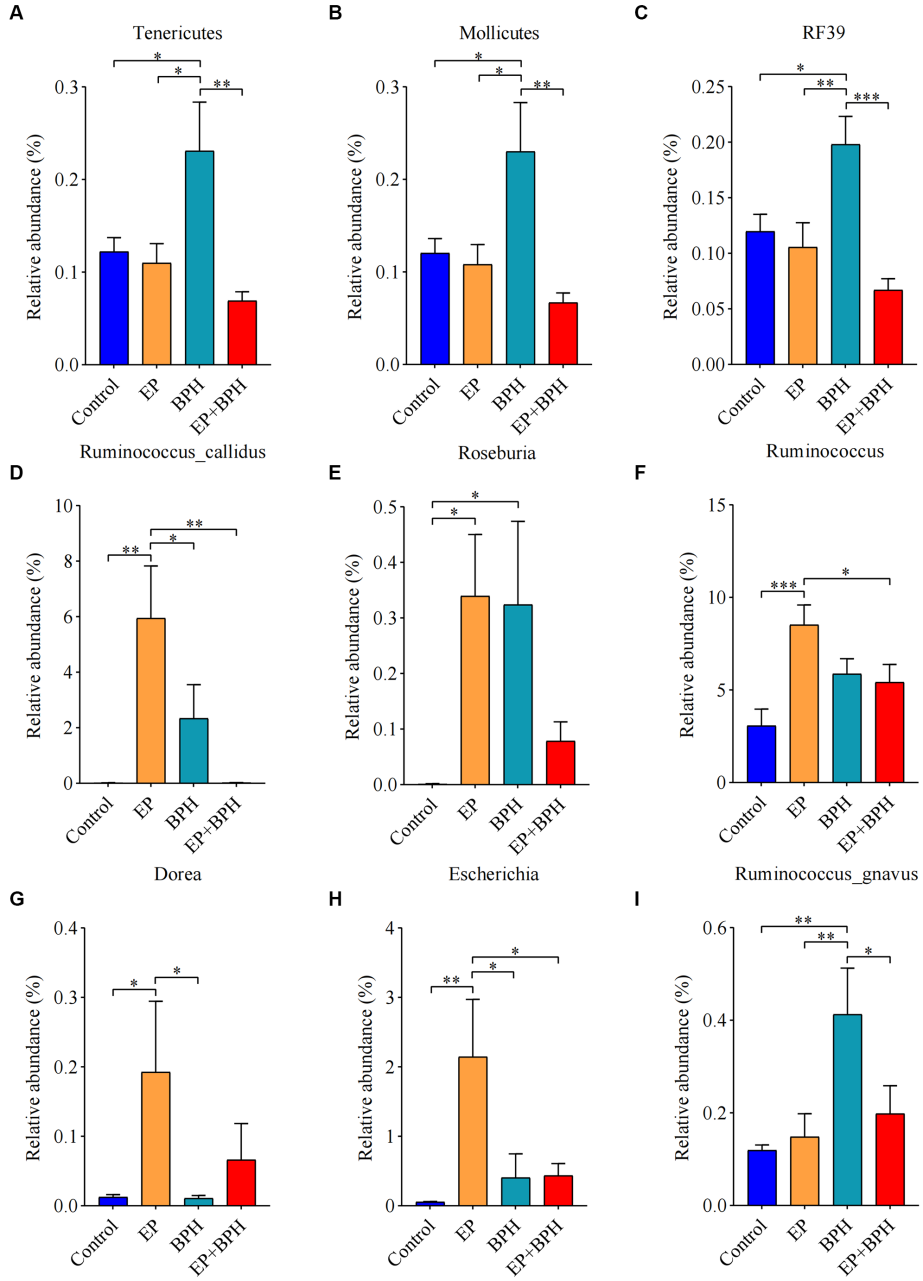
EP + BPH affects the abundance of gut microbiota, which is different from EP or BPH. Comparison of relative abundance of *Tenericutes* **(A)**, Mollicutes **(B)**, RF39 **(C)**, *Ruminococcus callidus* **(D)**, *Roseburia* **(E)**, *Ruminococcus* **(F)**, *Dorea* **(G)**, *Escherichia coli* **(H),** and *Ruminococcus gnavus* **(I)** in different groups. ^*^ *p* < 0.05, ^**^ *p* < 0.01, ^***^ *p* < 0.001.

To explain the alteration of gut microbiota structure and diversity in four groups, we detected microbial composition and abundance. At the phylum level, there were 11 annotated microbiota (Supplementary Figure S2A), and at the class level, there were a total of 19 annotated microbiota (Supplementary Figure S2B). At the family level, there were 20 annotated microbiota (Figure 2A), and at the genus level, there were a total of 22 annotated microbiota (Figure 2B). They showed the abundance of gut microbiota in four groups, with differences between groups. Compared with the control group, the abundance of *Tenericutes*, *Mollicutes,* and *RF39* was significantly increased in the BPH group, whereas it significantly decreased in the EP + BPH group when compared with the BPH group (Figures 3A–C). Compared with the control group, the abundance of *Ruminococcus callidus*, *Roseburia,* and *Ruminococcus* in the EP group and BPH group all tended to increase; the EP group increased more significantly, but the EP + BPH group had a more significant decrease compared with the EP group (Figures 3D–F). Compared with the control group, the abundance of *Dorea* and *Escherichia coli* in the EP group was significantly increased, while the two bacteria in the EP + BPH group were significantly decreased compared with the EP group (Figures 3G,H). Compared with the control group, the abundance of *Ruminococcus gnavus* in the EP, BPH, and EP + BPH groups showed an increasing trend, and the increase in the BPH group was more significant (Figure 3I), while compared with the BPH group, it decreased in the EP + BPH group (Figure 3I). Compared with the control group, the abundance of *Paraprevotella*, *Ruminococcus flavefaciens,* and *Prevotellaceae* in the EP, BPH, and EP + BPH groups showed an increasing trend, with the highest abundance in the EP + BPH group (Figure S3A–C), meanwhile, the abundance of *Porphyromonadaceae* and *Parabacteroides* also increased in four groups, especially in the EP + BPH group, although there was no statistical difference (*p* > 0.05; Supplementary Figures S3D,E). Compared with the control group, the abundance of *Erysipelotrichaceae* and *Peptostreptococcaceae* in the EP, BPH, and EP + BPH groups showed a decreasing trend, especially in the EP + BPH group (Supplementary Figures S3F,G). The above results suggest that the EP + BPH group elicited dramatic changes and complex linkages in the gut microbiota compared to the EP or BPH groups alone.

We use the KEGG database to analyze differences between groups of gut microbiota and predict functional pathways. A total of 68 pathways are regulated by EP and BPH. After comparing between groups, it was found that one pathway was specifically regulated by EP + BPH (EP + BPH/BPH and EP + BPH/EP), and neither EP (EP/Control) nor BPH (BPH/Control) alone had any regulatory effect on it. Simultaneously, we found that 29 pathways were jointly regulated by EP (EP/Control) and BPH (BPH/Control), but EP + BPH (EP + BPH/BPH or EP + BPH/EP) lost the regulation effect (Supplementary Figure S4). The above results indicated that gut microbiota function changed significantly in the EP + BPH group compared with the EP or BPH groups alone.

### 3.4 Overview of gut metabolome

**Figure 4:**
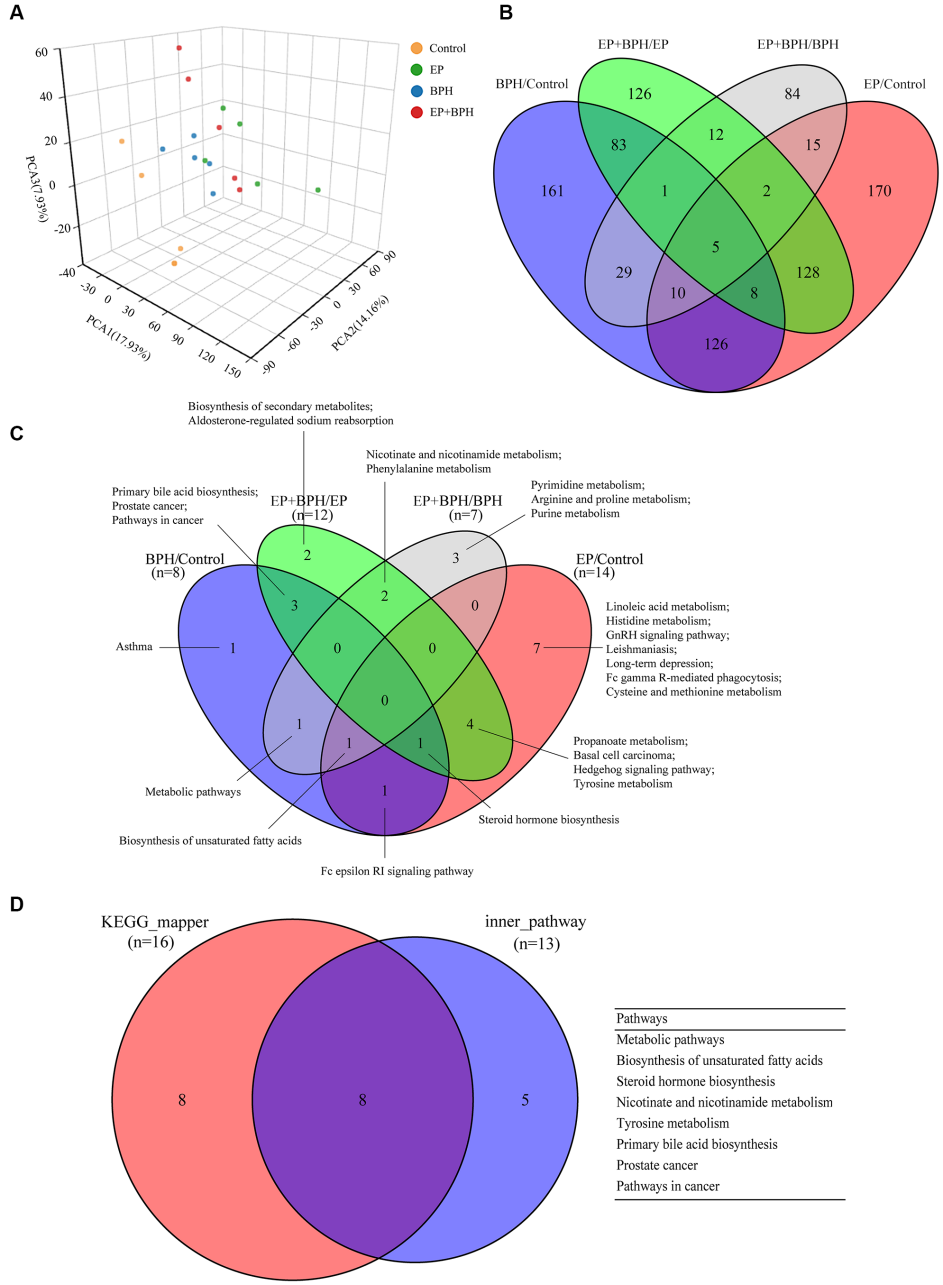
EP and BPH may affect fecal metabolites through interaction in rats. **(A)** Principal component analysis (PCA) for different groups: different colors represent different groups. Petal diagram of metabolites **(B)** and metabolic pathways **(C)** in different comparison pairs: different colors represent different pairs, and the overlapping parts of the numbers are common. **(D)** Intersection of different functional enrichment pathways.

We further analyzed the metabolites of rat feces using LC–MS to assess the effects of EP, BPH, and EP + BPH on the gut metabolome. PCA analysis revealed global metabolic changes in four groups of rats, and the results showed that the EP, BPH, and EP + BPH groups were all separated from the control group, and the EP + BPH group was also separated from the EP or BPH group (Figure 4A). After screening in the KEGG database, we found 960 fecal metabolites were regulated by EP and BPH. Comparing the four groups, 161 metabolites were specifically regulated by BPH (BPH/Control), 170 metabolites were specifically regulated by EP (EP/Control), 149 metabolites were regulated by both EP and BPH, and 12 metabolites were specifically regulated by EP + BPH (EP + BPH/EP and EP + BPH/BPH). The above results suggest that EP + BPH increases the regulation of fecal metabolites compared to EP or BPH alone (Figure 4B).

### 3.5 EP + BPH affects fecal metabolites and metabolic pathways

Compared with BPH/Control, Supplementary Figure S5A shows the effects of EP on fecal metabolites in the EP + BPH group. A total of 45 (6 upregulated, 5 downregulated, and 34 contraregulated) fecal metabolites were coregulated by EP and BPH, and 113 (51 upregulated, 62 downregulated) fecal metabolites were regulated by EP alone in the composite model (Supplementary Figure S5A). Figure 4B shows the effects of BPH on fecal metabolites in the EP + BPH group 143 (72 upregulated, 20 downregulated, and 5 contraregulated) fecal metabolites were coregulated by EP and BPH, and 222 (181 upregulated and 87 downregulated) fecal metabolites were regulated by BPH alone in the composite model (Supplementary Figure S5B). We used a heat map to compare the abundance of the above fecal metabolites (Supplementary Figure S6). When compared with the BPH or EP group alone, fecal metabolites are altered in the EP + BPH group, which further showed that EP or BPH may have an effect on fecal metabolites.

**Figure 5:**
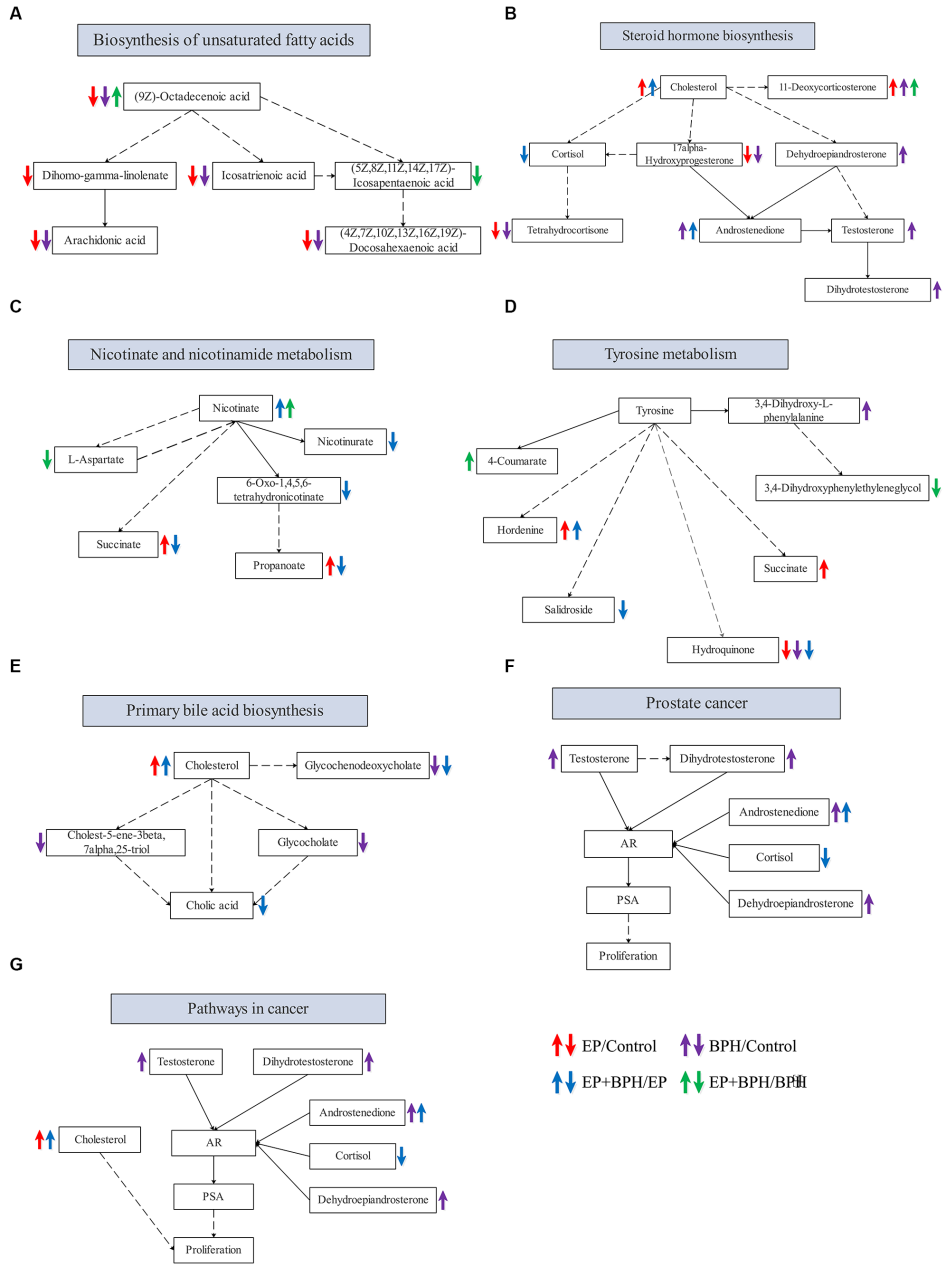
Alteration in metabolites in metabolic pathways that may be related to the interaction between EP and BPH. Alteration of related metabolites in the biosynthesis of unsaturated fatty acids **(A)**, steroid hormone biosynthesis **(B)**, nicotinate and nicotinamide metabolism **(C)**, tyrosine metabolism **(D)**, primary bile acid biosynthesis **(E)**, prostate cancer **(F),** and pathways in cancer **(G)**.

PEA identified 26 pathways regulated by EP and BPH (*p* < 0.05), including 3 specific to BPH (BPH/Control or EP + BPH/EP), 10 specific to EP (EP/Control or EP + BPH/BPH), and the remaining 13 intersection pathways (inner pathway) (Figure 4C). Additionally, 420 of the 960 regulatory metabolites had KEGG numbers and could be assigned to 104 metabolic pathways (Supplementary Table S1). Pathways of interest with ≥5 matched metabolites were selected (KEGG mapper), and then a total of eight effective pathways were found through crossover between the KEGG mapper pathway and the inner pathway from Figure 4C, as shown in the Venn diagram (Figure 4D). In addition to the metabolic pathway, we selected seven metabolic pathways for further analysis. Figure 5 shows the changes in metabolites in seven metabolic pathways in each comparison group.

### 3.6 Correlation between gut microbiota and fecal metabolites

**Figure 6:**
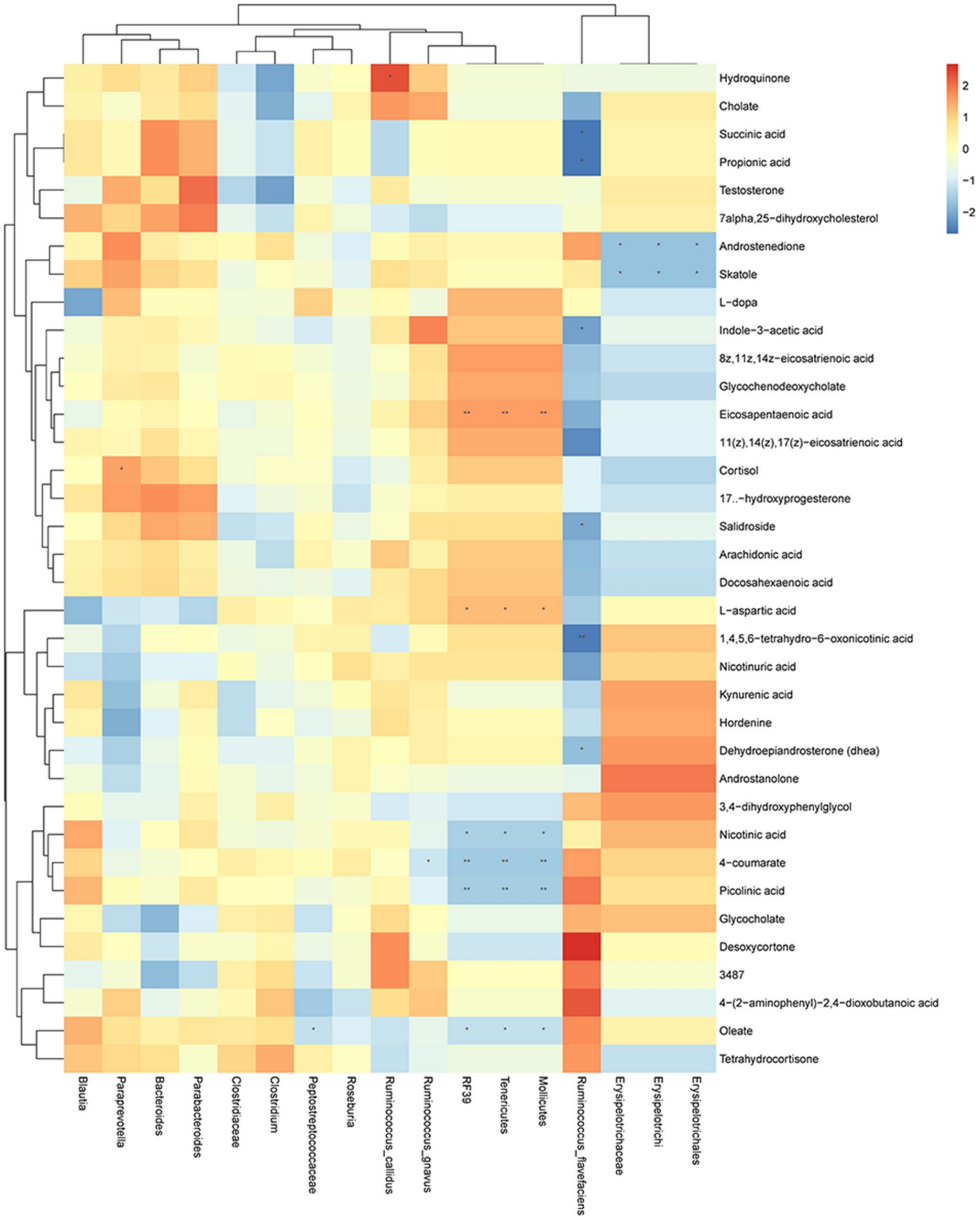
Changes in gut microbiota are correlated with the changes in metabolites. Heat map of correlations between gut microbiota and metabolites in metabolic pathways: X axis is the changed gut microbiota, and the Y axis is the metabolites in the metabolic pathway. ^*^ *p* < 0.05, ^**^ *p* < 0.01.

To further understand the relationship between gut microbiota and fecal metabolites, we performed a correlation analysis between gut microbiota and metabolites in seven metabolic pathways we focused on (Figure 6). Results showed that hydroquinone was significantly correlated with *Ruminococcus callidus*. Succinic acid, propionic acid, indole-3-acetic acid, salidroside 1,4,5,6-tetrahydro-6-oxonicotinic acid, and dehydroepiandrosterone were significantly correlated with *Ruminococcus flavefaciens*. Androstenedione and skatole were significantly correlated with *Erysipelotrichi*, *Erysipelotrichaceae,* and *Erysipelotrichales*. Eicosapentaenoic acid, L-aspartic acid, nicotinic acid, 4-coumarate, picolinic acid, and oleate were significantly correlated with *Mollicutes*, *Tenericutes,* and *RF39*. 4-coumarate was significantly correlated with *Ruminococcus gnavus*. Oleate was significantly correlated with *Peptostreptococcaceae*. Cortisol was significantly correlated with *Paraprevotella*.

## 4 Discussion

Periodontitis and BPH are two common age-related diseases with a high prevalence. Studies have shown a link between periodontitis and BPH (Boland et al., 2013; Estemalik et al., 2017; Fu et al., 2021), and we have previously demonstrated that periodontal disease is positively associated with an increased risk of BPH (Wu et al., 2019); and periodontitis could promote the development of BPH through the regulation of oxidative stress and inflammation (Fang et al., 2021). Both periodontitis and BPH have been proven to be related to gut microbiota (Albuquerque-Souza and Sahingur, 2022; Russo et al., 2022). In this study, we found periodontitis and BPH may interact with each other to affect gut microbiota and metabolites.

We found that the diversity of gut microbiota in EP + BPH was different from other groups. Gut microbiota, such as *Paraprevotella*, *Ruminococcus flavefaciens*, *Peptostreptococcaceae,* and *Ruminococcus gnavus*, have significantly changed in the EP + BPH group. The fecal metabolites and metabolic pathways, such as the biosynthesis of unsaturated fatty acids and steroid hormone biosynthesis, have also changed in the EP + BPH group. These results suggest that BPH and EP may interact with each other to affect the gut microbiota and metabolism. Correlation analysis showed that changes in metabolites are correlated with the gut microbiota.

For *Escherichia coli*, the abundance was significantly increased in the EP group compared with the control group, but insignificantly increased in the BPH and EP + BPH groups (Supplementary Figure S4H). Estemalik et al. have detected *Escherichia coli* in dental plaque and prostatic secretion samples from periodontitis patients with prostatic hyperplasia/inflammation through RT-PCR (Estemalik et al., 2017). It has been suggested that the etiology of BPH is due to repeated colonization followed by *Escherichia coli* destruction in the prostatic transition zone (Roper, 1998, 2017). When *Escherichia coli* are destroyed, phospholipase D is released and affects prostate cells (Roper, 2017). Jain et al. have found that *Escherichia coli* could activate nuclear factor kappa-B and induce DNA damage in prostate epithelial cells (Jain et al., 2020). Furthermore, the abundance of *Ruminococcus* was significantly altered in our study. Henke et al. have found that *Ruminococcus gnavus* synthesizes and secretes a complex glucorhamnan, which could induce inflammatory cytokine (TNFα) secretion by dendritic cells (Henke et al., 2019). A recent study reported that it could convert androgen precursors into active androgens to promote the development of prostate cancer (Henke et al., 2019), and *Ruminococcus* could also promote the progression of prostate cancer by upregulating the expression of LPCAT1 and DNA repair proteins in prostate tissues (Liu et al., 2021). Our result suggests BPH and EP may jointly alter the gut microbiota, *Escherichia coli,* and *Ruminococcus* may play an important role in the development of BPH and EP and their interactions. In our previous study, we proposed an oral–prostate axis hypothesis, an oral–genitourinary axis, and gut–genitourinary axis (Fang et al., 2021; Yuan et al., 2021). It has been suggested that the host has an oral–gut axis, which plays an important role in regulating the pathological processes of diseases (Kitamoto et al., 2020; Park et al., 2021). So we hypothesized that there could be an oral–gut–prostate axis.

Figure 5 shows metabolic pathways enriched in the EP + BPH group. Hutcherson et al. (2016) have reported that the nicotinate and nicotinamide metabolism pathways may be involved in the process of *Porphyromonas gingivalis* causing periodontal disease. The metabolic pathway in cancer may be involved in the pathological process of peri-implantitis (Zhang et al., 2021). Our previous study also found that tyrosine metabolism may be associated with both periodontitis and BPH (Li et al., 2022; Wu et al., 2022). In addition, the biosynthesis of unsaturated fatty acids, steroid hormone biosynthesis, and the primary bile acid biosynthesis pathway may play an important role in the development of BPH (Li et al., 2022). Therefore, combined with our previous study, we think that the body may mediate the interaction between EP and BPH diseases through the above pathways.

Correlation analysis showed that metabolites involved in metabolic pathways were significantly correlated with the gut microbiota, especially *Ruminococcus flavefaciens*, *Mollicutes*, *Tenericutes,* and *RF39* (Figure 6). *Mollicutes* have been found in dental plaque and may be associated with periodontitis (Hutter et al., 2003; Shiba et al., 2021). Our previous study also found a correlation between *Mollicutes* and BPH (Li et al., 2022), suggesting that *Mollicutes* may mediate the interaction between BPH and EP. *Tenericutes*, as a representative bacteria in the oral microbiota (Wade, 2013), may be associated with periodontal disease (Vengerfeldt et al., 2014; Zhang et al., 2020). Miranda-Rius et al. (2021) have reported that *Tenericutes* may also be associated with adverse pregnancy, suggesting that periodontal disease may have an impact on other systems, such as the urinary system and gastrointestinal tract, through *Tenericutes*. These evidence suggest that *Mollicutes* and *Tenericutes* play an important role in the interaction between BPH and EP.

Gut microbiota and host have a close symbiotic relationship, and they could appear to have a bidirectional impact. Studies have found that the imbalance of the microbiota is involved in the pathogenesis of many diseases, such as cardiovascular disease, colon cancer, Crohn’s disease, diabetes, irritable bowel syndrome, obesity, hepatic encephalopathy, mental disorders, and many other diseases (Lynch and Pedersen, 2016; Young, 2017; Witkowski et al., 2020; Hou et al., 2022; Won et al., 2022; Crudele et al., 2023; Wong and Yu, 2023). Gut microbiota can cause diarrhea, gastrointestinal inflammation, and the malignant progression of colon cancer through direct or indirect action (Garrett, 2019). It could also affect the host by altering intestinal metabolites. Experiments have shown that gut microbiota increases appetite by affecting the level of acetate in the gut, which in turn increases ghrelin secretion and enhances fat storage by promoting insulin secretion (Perry et al., 2016). Gut microbiota also induce metabolic reprogramming of osteoclasts, resulting in enhanced glycolysis at the expense of oxidative phosphorylation and regulating osteoclasts by downregulating TRAF6 and NFATc1, thereby affecting bone homeostasis (Lucas et al., 2018). Various experimental studies in germ-free animals have shown that gut microbes promote carcinogenic effects in various organs, such as the lungs, skin, and breasts (Illiano et al., 2020).

The microbiota is also affected by factors such as health status, diet, drugs, medications, and lifestyle habits (Fan and Pedersen, 2021). Most people in developed countries have a lower diversity and number of gut microbes than people in East Africa or the Amazon (Fan and Pedersen, 2021). Obesity is associated with alteration in gut microbiota; a high-fat diet could reduce gut bacteria density, increase the relative proportion of *Bacteroidales* and *Clostridiales*, and affect the diversity of the microbiota (de La Serre et al., 2010; Beam et al., 2021; Tong et al., 2021). Patients with type 2 diabetes have dysbiosis, and the markers of the gut microbiota could be used to classify type 2 diabetes (Wang et al., 2017; Gurung et al., 2020). The study investigated the relationship between different drugs and the abundance of the gut microbiota, as well as their relationship to disease severity, and revealed the drug–host–microbiome association in which patients taking drugs to treat disease can significantly affect the gut microbiota (Forslund et al., 2021). Clinical studies have shown that people with obesity and type 2 diabetes have altered microbiota composition, suggesting that metabolism-related diseases may affect the composition of the gut microbiota (Karlsson et al., 2013; Illiano et al., 2020). Many studies have confirmed bidirectional interactions among the brain, the gut, and the gut microbiota. The gut–brain axis has clearly been established in irritable bowel syndrome (Ford et al., 2020; Mayer et al., 2022). The above evidence suggests that there is a bidirectional relationship between gut microbiota and disease. Therefore, it is of great significance for us to further understand the gut microbiota and its related diseases to find out the relevant factors affecting the intestinal microbiota.

## 5 Conclusion

We found that EP and BPH may interact with each other, and alteration of gut microbiota and metabolites is associated with the interaction of the two diseases, which lays the foundation for further research and provides a new idea for the prevention and treatment of patients with periodontitis concurrent with BPH.

## Data availability statement

The datasets presented in this study can be found in online repositories; https://www.ncbi.nlm.nih.gov/, PRJNA762590, PRJNA778630, PRJNA880705.

## Ethics statement

The animal study was approved by the Institutional Animal Care and Use Committee (IACUC) of Wuhan University Wuhan University. The study was conducted in accordance with the local legislation and institutional requirements.

## Author contributions

X-PG: Writing – original draft, Investigation. JY: Writing – original draft. LW: Conceptualization, Investigation, Writing – review & editing. CF: Writing – review & editing, Methodology, Software. J-MG: Writing – review & editing, Investigation. FL: Investigation, Writing – review & editing. H-SL: Writing – review & editing, Conceptualization. L-YL: Conceptualization, Writing – original draft. S-YW: Project administration, Software, Visualization, Writing – review & editing.
