# Supplementary material for: Periodontitis relates to benign prostatic hyperplasia via the gut microbiota and fecal metabolome
Source: Front Microbiol. 2023 Dec 13;14:1280628. doi: 10.3389/fmicb.2023.1280628 (PMC10756679; doi:10.3389/fmicb.2023.1280628)
Supplement: Supplementary file 1 [file Data_Sheet_1.docx]

Supplementary Material

**Supplementary Tables and Figures**

# Supplementary Tables

**Table S1**

**Relevant pathways matched with the differentially regulated metabolites in KEGG mapper**

| Pathway_ID | KEGG_pathway | Number | HMDB_name of regulated metabolites |
| --- | --- | --- | --- |
| hsa01100 | Metabolic pathways | 82 | 4-Hydroxy-2-quinolinecarboxylic acid; Glycocholate; Pseudouridine; Protoporphyrin; N-Acetylmuramate; L-Methionine S-oxide; 11-Deoxycorticosterone; (E)-2-Methylpropanal oxime; Dihomo-gamma-linolenate; Dihydro-O-methylsterigmatocystin; 5-(2-Hydroxyethyl)-4-methylthiazole; Phenethylamine; D-Glucuronate 1-phosphate; Vitamin D2; Deoxyinosine; 3,4-Dihydroxyphenylethyleneglycol; Phenylpropanoate; Eriodictyol; D-Urobilin; Kaempferol; Salidroside; Adipate; Hordenine; Tetradecanoic acid; Naringenin chalcone; Erythronolide B; Styrene; Crocetin; Isoliquiritigenin; Cyclohexane-1-carboxylate; Picolinic acid; Daidzein; Gibberellin A12; Oleandolide; 10-Deoxymethymycin; 1,2-Dihydroxy-5-(methylthio)pent-1-en-3-one; 7-Mercaptoheptanoylthreonine; Aflatoxin G2 |
| hsa00140 | Steroid hormone biosynthesis | 9 | Cholesterol; Androstenedione; Testosterone; Cortisol; 17alpha-Hydroxyprogesterone; Dehydroepiandrosterone; 11-Deoxycorticosterone; Dihydrotestosterone; Tetrahydrocortisone |
| hsa02010 | ABC transporters | 9 | L-Aspartate; L-Histidine; Nitrate; Inosine; Uridine; Raffinose; Deoxyuridine; Deoxyinosine; Mannopine |
| hsa04976 | Bile secretion | 8 | Cholesterol; Urate; Cholic acid; Cortisol; Spermine; Glycocholate; Glycochenodeoxycholate; Tenofovir (USAN) |
| hsa00350 | Tyrosine metabolism | 7 | Succinate; 3,4-Dihydroxy-L-phenylalanine; Hydroquinone; 4-Coumarate; 3,4-Dihydroxyphenylethyleneglycol; Salidroside; Hordenine |
| hsa01240 | Biosynthesis of cofactors | 6 | L-Aspartate; Nicotinate; Pyridoxine; Spermine; Protoporphyrin; 5-(2-Hydroxyethyl)-4-methylthiazole |
| hsa00760 | Nicotinate and nicotinamide metabolism | 6 | Succinate; L-Aspartate; Propanoate; Nicotinate; 6-Oxo-1,4,5,6-tetrahydronicotinate; Nicotinurate |
| hsa05200 | Pathways in cancer | 6 | Cholesterol; Androstenedione; Testosterone; Cortisol; Dehydroepiandrosterone; Dihydrotestosterone |
| hsa04913 | Ovarian steroidogenesis | 6 | Cholesterol; Arachidonate; Androstenedione; Testosterone; 17alpha-Hydroxyprogesterone; Dehydroepiandrosterone |
| hsa04080 | Neuroactive ligand-receptor interaction | 6 | L-Aspartate; Histamine; Cortisol; Anandamide; 2-Arachidonoylglycerol; Palmitoylethanolamide |
| hsa01040 | Biosynthesis of unsaturated fatty acids | 6 | Arachidonate; (9Z)-Octadecenoic acid; Dihomo-gamma-linolenate; (5Z,8Z,11Z,14Z,17Z)-Icosapentaenoic acid; (4Z,7Z,10Z,13Z,16Z,19Z)-Docosahexaenoic acid; Icosatrienoic acid |
| hsa00380 | Tryptophan metabolism | 5 | Indole-3-acetate; 4-(2-Aminophenyl)-2,4-dioxobutanoate; 4-Hydroxy-2-quinolinecarboxylic acid; Skatole; Picolinic acid |
| hsa00120 | Primary bile acid biosynthesis | 5 | Cholesterol; Cholic acid; Glycocholate; Glycochenodeoxycholate; Cholest-5-ene-3beta,7alpha,25-triol |
| hsa05215 | Prostate cancer | 5 | Androstenedione; Testosterone; Cortisol; Dehydroepiandrosterone; Dihydrotestosterone |
| hsa00240 | Pyrimidine metabolism | 5 | Thymine; Thymidine; Uridine; Deoxyuridine; Pseudouridine |
| hsa00270 | Cysteine and methionine metabolism | 5 | L-Aspartate; 5'-Methylthioadenosine; S-Adenosylmethioninamine; L-Methionine S-oxide; 1,2-Dihydroxy-5-(methylthio)pent-1-en-3-one |
| hsa00360 | Phenylalanine metabolism | 4 | Succinate; trans-Cinnamate; Phenethylamine; Phenylpropanoate |
| hsa00230 | Purine metabolism | 4 | Inosine; dAMP; Urate; Deoxyinosine |
| hsa01210 | 2-Oxocarboxylic acid metabolism | 4 | L-Aspartate; N-Acetyl-L-glutamate; (E)-2-Methylpropanal oxime; 3-(6'-Methylthio)hexylmalic acid |
| hsa00340 | Histidine metabolism | 4 | L-Aspartate; L-Histidine; Histamine; 4-(beta-Acetylaminoethyl)imidazole |
| hsa04974 | Protein digestion and absorption | 4 | L-Aspartate; L-Histidine; Propanoate; Histamine |
| hsa01230 | Biosynthesis of amino acids | 4 | L-Aspartate; L-Histidine; L-Citrulline; N-Acetyl-L-glutamate |
| hsa00330 | Arginine and proline metabolism | 3 | Spermine; Creatinine; S-Adenosylmethioninamine |
| hsa04927 | Cortisol synthesis and secretion | 3 | Cholesterol; Cortisol; 17alpha-Hydroxyprogesterone |
| hsa04723 | Retrograde endocannabinoid signaling | 3 | Arachidonate; Anandamide; 2-Arachidonoylglycerol |
| hsa04750 | Inflammatory mediator regulation of TRP channels | 3 | Arachidonate; Histamine; Anandamide |
| hsa05230 | Central carbon metabolism in cancer | 3 | Succinate; L-Aspartate; L-Histidine |
| hsa04979 | Cholesterol metabolism | 3 | Cholesterol; Glycocholate; Glycochenodeoxycholate |
| hsa00860 | Porphyrin metabolism | 3 | Protoporphyrin; I-Urobilinogen; D-Urobilin |
| hsa00130 | Ubiquinone and other terpenoid-quinone biosynthesis | 3 | trans-Cinnamate; 4-Coumarate; Deoxyshikonin |
| hsa04934 | Cushing syndrome | 3 | Cholesterol; Cortisol; 17alpha-Hydroxyprogesterone |
| hsa01200 | Carbon metabolism | 3 | Succinate; L-Aspartate; D-Glucono-1,5-lactone |
| hsa00410 | beta-Alanine metabolism | 3 | L-Aspartate; L-Histidine; Spermine |
| hsa00220 | Arginine biosynthesis | 3 | L-Aspartate; L-Citrulline; N-Acetyl-L-glutamate |
| hsa04925 | Aldosterone synthesis and secretion | 3 | Cholesterol; Arachidonate; 11-Deoxycorticosterone |
| hsa00250 | Alanine, aspartate and glutamate metabolism | 2 | Succinate; L-Aspartate |
| hsa00440 | Phosphonate and phosphinate metabolism | 2 | D-Ribose 1,5-bisphosphate; Rhizocticin A |
| hsa00980 | Metabolism of xenobiotics by cytochrome P450 | 2 | 1,4-Naphthoquinone; S-[2-(N7-Guanyl)ethyl]-N-acetyl-L-cysteine |
| hsa00100 | Steroid biosynthesis | 2 | Cholesterol; Vitamin D2 |
| hsa00520 | Amino sugar and nucleotide sugar metabolism | 2 | N-Acetylmuramate; D-Glucuronate 1-phosphate |
| hsa00061 | Fatty acid biosynthesis | 2 | (9Z)-Octadecenoic acid; Tetradecanoic acid |
| hsa04714 | Thermogenesis | 2 | Anandamide; 2-Arachidonoylglycerol |
| hsa01250 | Biosynthesis of nucleotide sugars | 2 | N-Acetylmuramate; D-Glucuronate 1-phosphate |
| hsa00030 | Pentose phosphate pathway | 2 | D-Glucono-1,5-lactone; D-Ribose 1,5-bisphosphate |
| hsa00640 | Propanoate metabolism | 2 | Succinate; Propanoate |
| hsa00770 | Pantothenate and CoA biosynthesis | 2 | L-Aspartate; Spermine |
| hsa00470 | D-Amino acid metabolism | 2 | L-Aspartate; L-Histidine |
| hsa04742 | Taste transduction | 2 | "Salicin; Aspartame |
| " |  |  |  |
| hsa04664 | Fc epsilon RI signaling pathway | 2 | Arachidonate; Histamine |
| hsa04024 | cAMP signaling pathway | 2 | Succinate; Oleoylethanolamide |
| hsa01522 | Endocrine resistance | 2 | Androstenedione; Testosterone |
| hsa05208 | Chemical carcinogenesis - reactive oxygen species | 2 | Hydroquinone; Benzene |
| hsa04917 | Prolactin signaling pathway | 2 | Androstenedione; 3,4-Dihydroxy-L-phenylalanine |
| hsa00591 | Linoleic acid metabolism | 2 | Arachidonate; Dihomo-gamma-linolenate |
| hsa00970 | Aminoacyl-tRNA biosynthesis | 2 | L-Aspartate; L-Histidine |
| hsa00052 | Galactose metabolism | 1 | Raffinose |
| hsa04726 | Serotonergic synapse | 1 | Arachidonate |
| hsa04971 | Gastric acid secretion | 1 | Histamine |
| hsa04912 | GnRH signaling pathway | 1 | Arachidonate |
| hsa04727 | GABAergic synapse | 1 | Succinate |
| hsa00790 | Folate biosynthesis | 1 | Neopterin |
| hsa00630 | Glyoxylate and dicarboxylate metabolism | 1 | Succinate |
| hsa00040 | Pentose and glucuronate interconversions | 1 | D-Glucuronate 1-phosphate |
| hsa04721 | Synaptic vesicle cycle | 1 | Histamine |
| hsa05310 | Asthma | 1 | Histamine |
| hsa00750 | Vitamin B6 metabolism | 1 | Pyridoxine |
| hsa00260 | Glycine, serine and threonine metabolism | 1 | L-Aspartate |
| hsa04977 | Vitamin digestion and absorption | 1 | Cholesterol |
| hsa00480 | Glutathione metabolism | 1 | Spermine |
| hsa04975 | Fat digestion and absorption | 1 | Cholesterol |
| hsa05417 | Lipid and atherosclerosis | 1 | Cholesterol |
| hsa04217 | Necroptosis | 1 | Arachidonate |
| hsa00190 | Oxidative phosphorylation | 1 | Succinate |
| hsa00310 | Lysine degradation | 1 | Succinate |
| hsa04921 | Oxytocin signaling pathway | 1 | Arachidonate |
| hsa00730 | Thiamine metabolism | 1 | 5-(2-Hydroxyethyl)-4-methylthiazole |
| hsa04922 | Glucagon signaling pathway | 1 | Succinate |
| hsa04960 | Aldosterone-regulated sodium reabsorption | 1 | Cortisol |
| hsa04923 | Regulation of lipolysis in adipocytes | 1 | Arachidonate |
| hsa04929 | GnRH secretion | 1 | Testosterone |
| hsa05030 | Cocaine addiction | 1 | 3,4-Dihydroxy-L-phenylalanine |
| hsa00620 | Pyruvate metabolism | 1 | Succinate |
| hsa00020 | Citrate cycle (TCA cycle) | 1 | Succinate |
| hsa05031 | Amphetamine addiction | 1 | 3,4-Dihydroxy-L-phenylalanine |
| hsa00920 | Sulfur metabolism | 1 | Succinate |
| hsa04611 | Platelet activation | 1 | Arachidonate |
| hsa00650 | Butanoate metabolism | 1 | Succinate |
| hsa05140 | Leishmaniasis | 1 | Arachidonate |
| hsa04270 | Vascular smooth muscle contraction | 1 | Arachidonate |
| hsa00910 | Nitrogen metabolism | 1 | Nitrate |
| hsa04973 | Carbohydrate digestion and absorption | 1 | Propanoate |
| hsa04261 | Adrenergic signaling in cardiomyocytes | 1 | Isoproterenol |
| hsa05217 | Basal cell carcinoma | 1 | Cholesterol |
| hsa04666 | Fc gamma R-mediated phagocytosis | 1 | Arachidonate |
| hsa00010 | Glycolysis / Gluconeogenesis | 1 | Salicin |
| hsa05133 | Pertussis | 1 | Nicotinate |
| hsa05146 | Amoebiasis | 1 | Arachidonate |
| hsa00053 | Ascorbate and aldarate metabolism | 1 | D-Glucuronate 1-phosphate |
| hsa04216 | Ferroptosis | 1 | Arachidonate |
| hsa04730 | Long-term depression | 1 | Arachidonate |
| hsa00590 | Arachidonic acid metabolism | 1 | Arachidonate |
| hsa05034 | Alcoholism | 1 | 3,4-Dihydroxy-L-phenylalanine |
| hsa05012 | Parkinson disease | 1 | 3,4-Dihydroxy-L-phenylalanine |

Totally 420 differentially regulated metabolites had KEGG numbers, which were input as a whole in KEGG mapper online. KEGG, Kyoto Encyclopedia of Genes and Genomes; HMDB, the Human Metabolome Database.

# Supplementary Figures


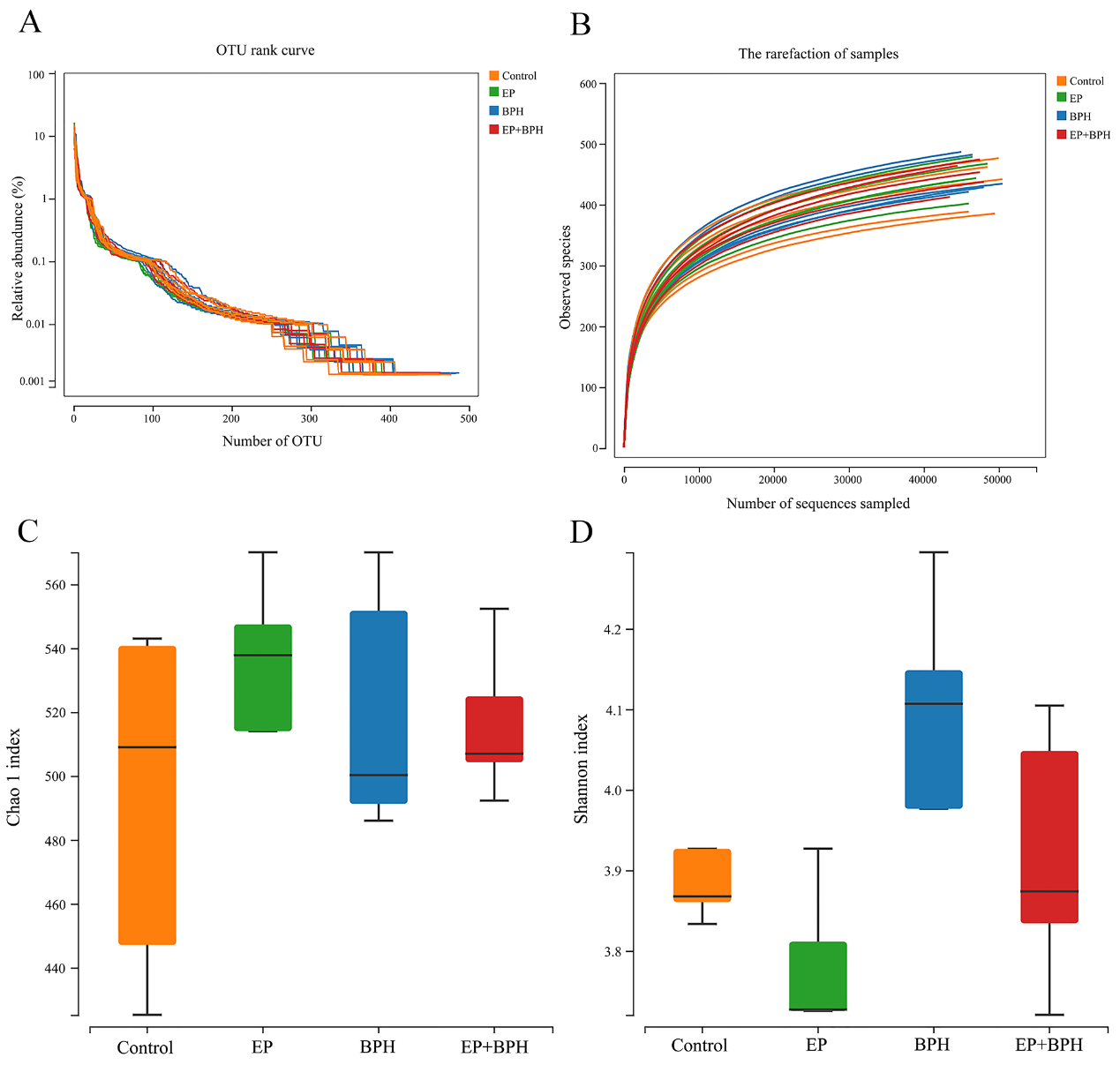


**Supplementary Figure 1** The sequencing evenness and richness of all groups reached the standard **(A)** OUT rank curve for each group. **(B)** Sample dilution curves for each group. Chao 1 index **(C)** and Shannon index **(D)** for each group.


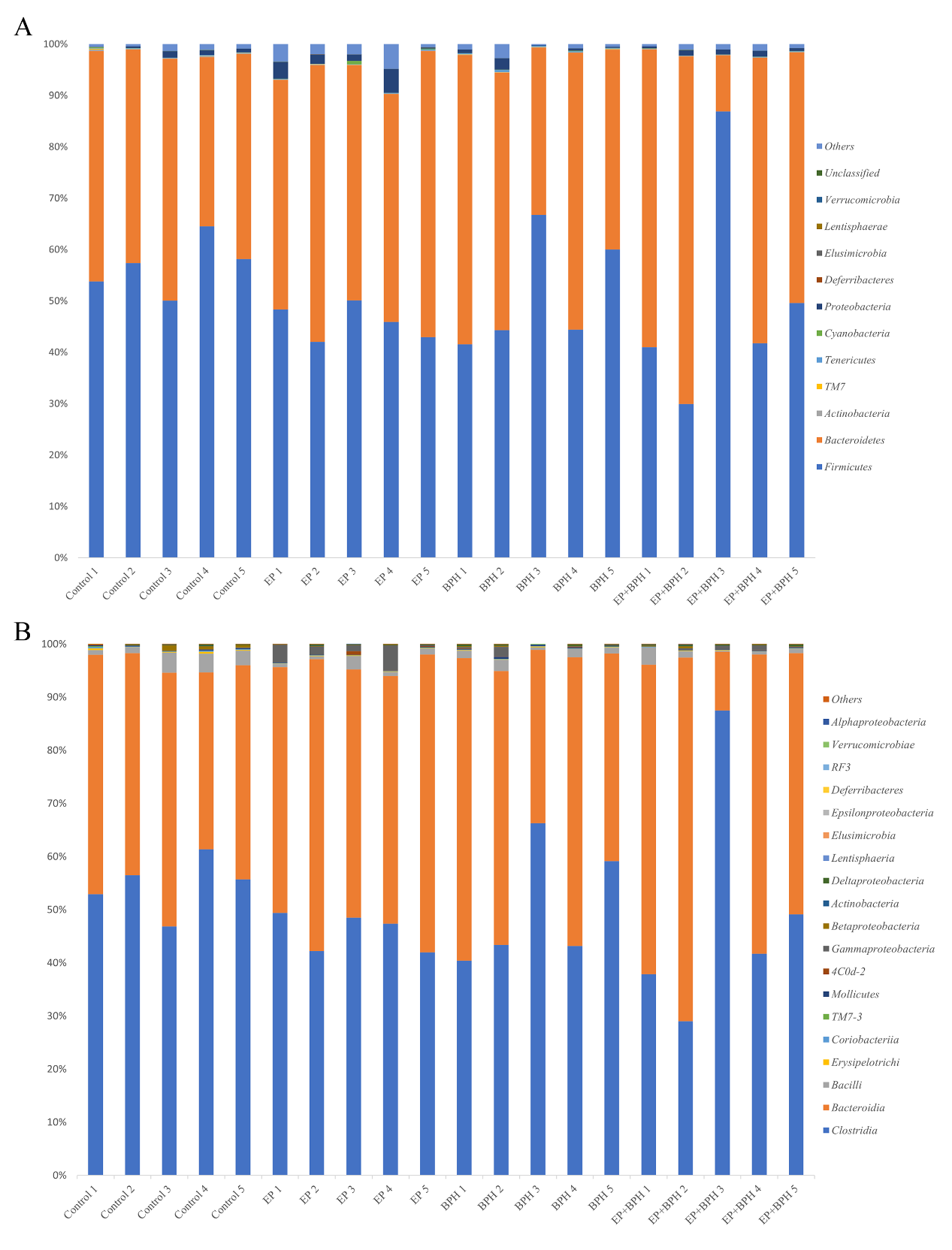


**Supplementary Figure 2** EP+BPH affects the structure of gut microbiota (associated with Figure 3)

The species bar chart shows the species composition and proportion of each sample at phylum level **(A)** and class level **(B)**. The X axis is the sample name and the Y axis is the relative abundance of annotated microbiota species.


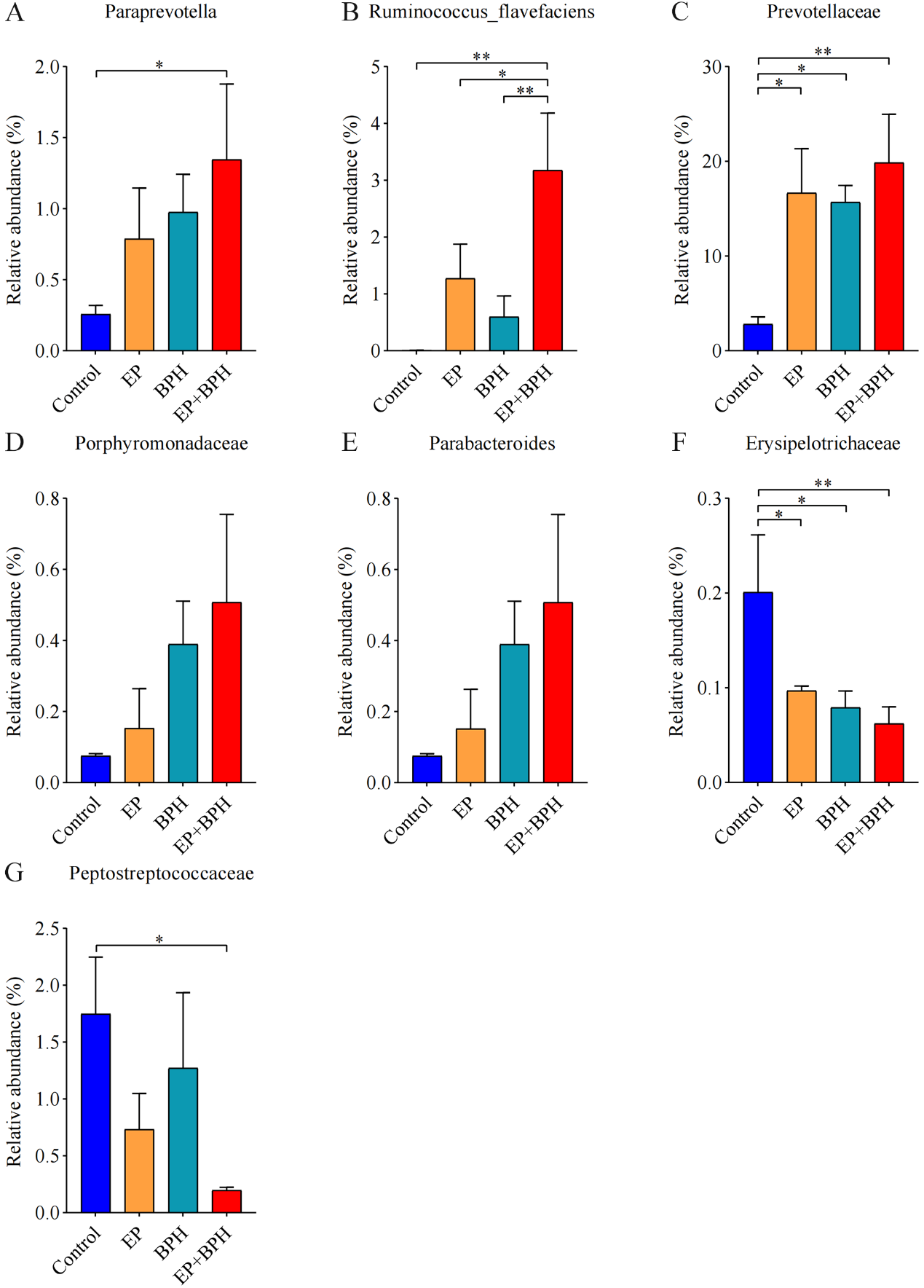


**Supplementary Figure 3** EP+BPH affects the abundance of gut microbiota (associated with Figure 3)The change of *Paraprevotella* **(A)**, *Ruminococcus flavefaciens* **(B)**, *Prevotellaceae* **(C)**, *Porphyromonadaceae* **(D)**, *Parabacteroides* **(E)** and *Erysipelotrichaceae* **(F)**.

^*^ *p* < 0.05, ^**^ *p* < 0.01.


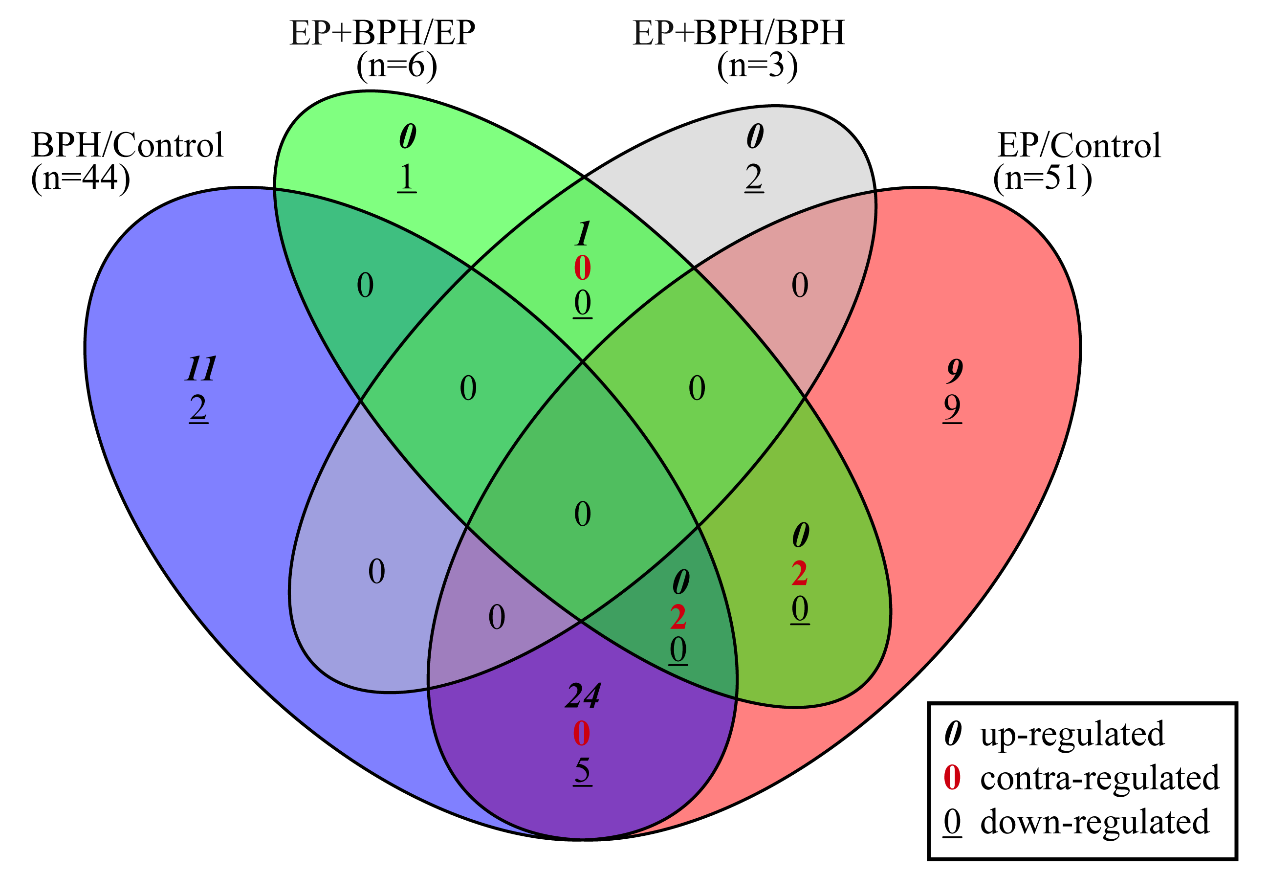


**Supplementary Figure 4** Changes of Gut microbiota KEGG pathway

Changes (up-regulated, contra-regulated and down-regulated) of KEGG pathway between different comparison groups.


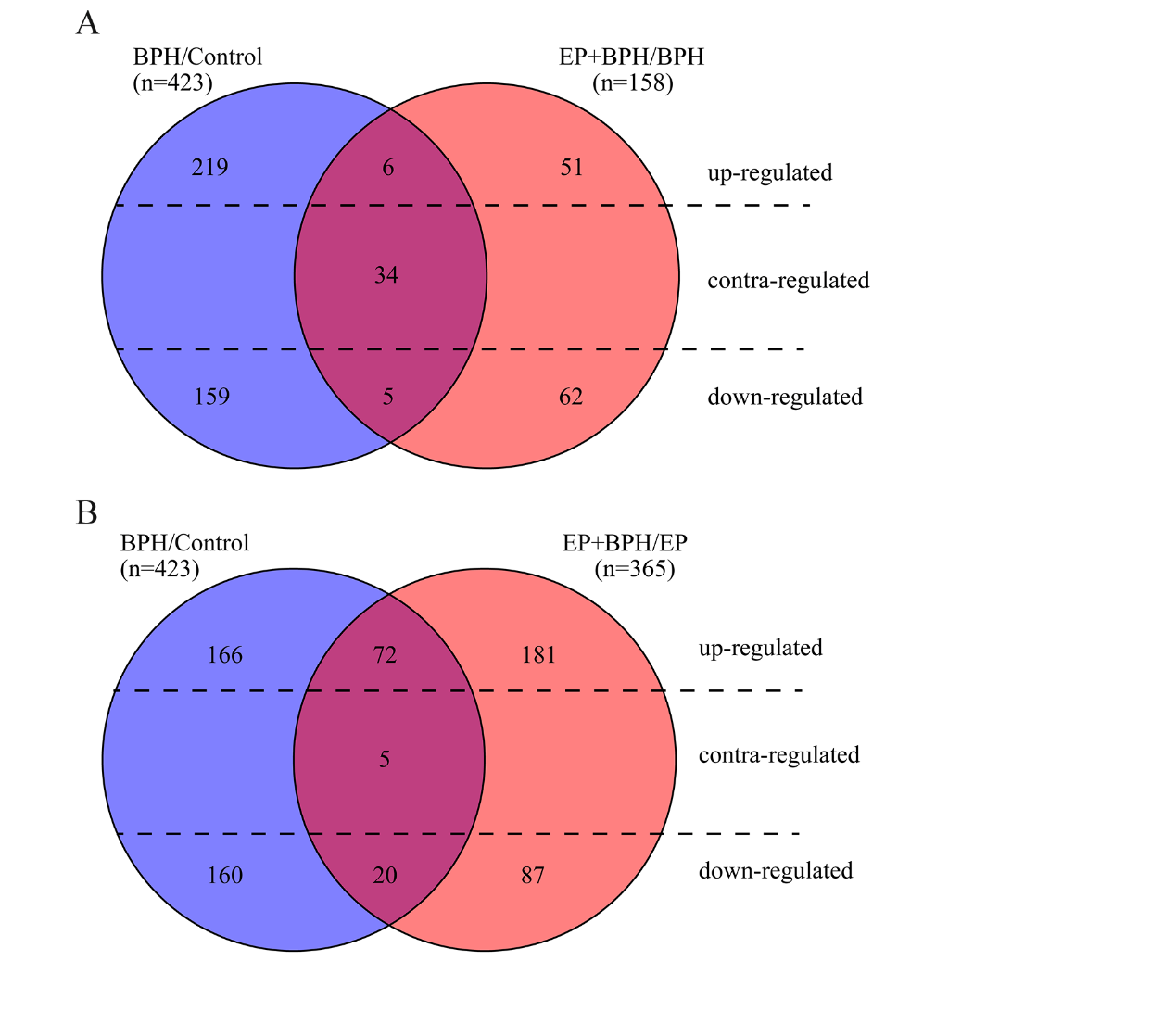


**Supplementary Figure 5** Effects of EP+BPH on fecal metabolites based on EP or BPH in rats

**(A)** Effects of EP+BPH on fecal metabolites based on BPH. **(B)** Effects of EP+BPH on fecal metabolites based on EP.


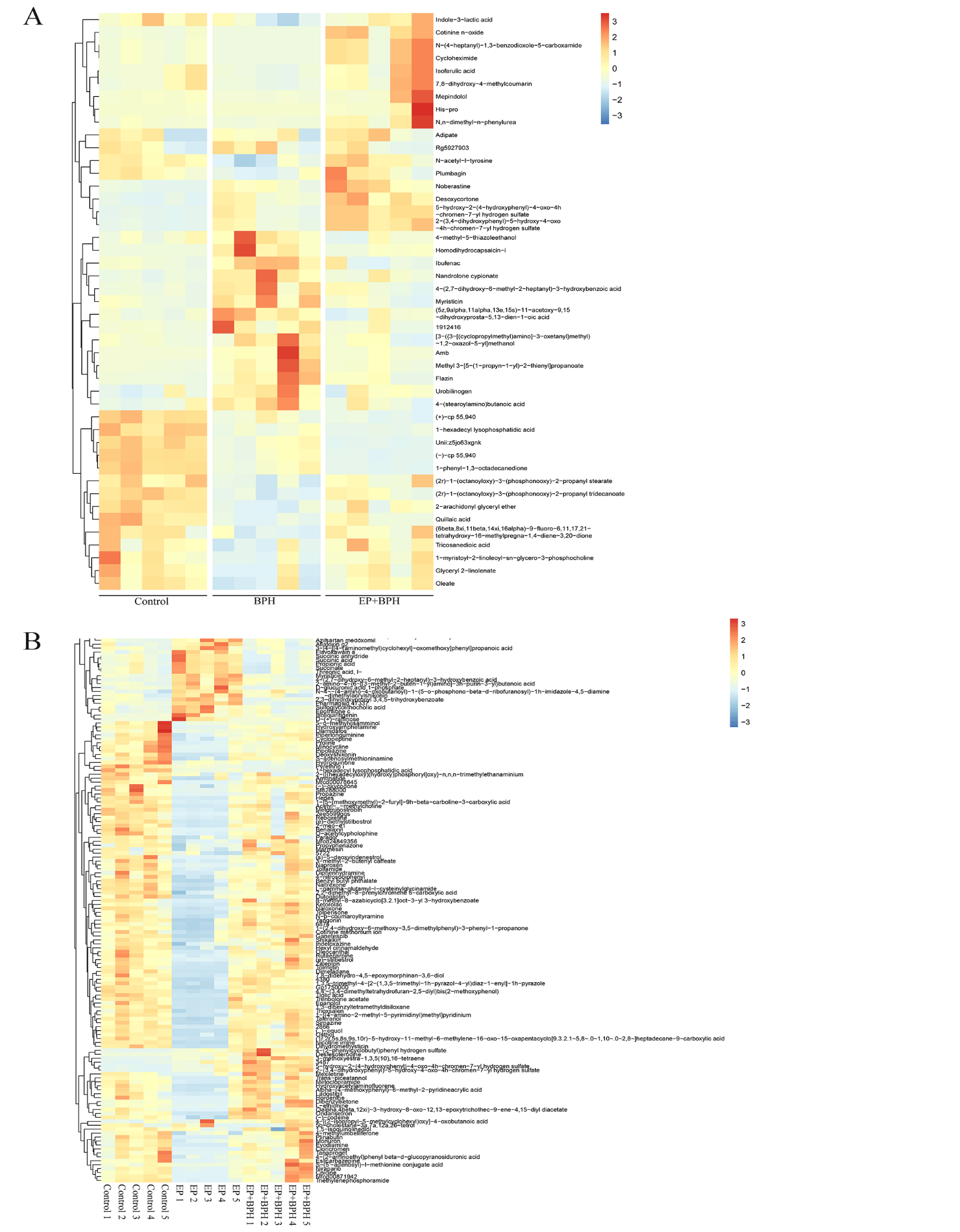
**Supplementary Figure 6** Heat map of effects of EP+BPH on fecal metabolites in EP or BPH rats

Heat map of effects of EP+BPH on fecal metabolites in BPH rats **(A)** and EP rats **(B)**, the X-axis represents the group, and the Y-axis represents the metabolite
